# Diagnostic properties of the Italian ECAS Carer Interview (ECAS-CI)

**DOI:** 10.1007/s10072-022-06505-x

**Published:** 2022-11-23

**Authors:** Barbara Poletti, Edoardo Nicolò Aiello, Federica Solca, Silvia Torre, Laura Carelli, Roberta Ferrucci, Federico Verde, Nicola Ticozzi, Vincenzo Silani

**Affiliations:** 1 Department of Neurology and Laboratory of Neuroscience, IRCCS Istituto Auxologico Italiano, Milano, Italy; 2 PhD Program in Neuroscience, School of Medicine and Surgery, University of Milano-Bicocca, Monza, Italy; 3 Aldo Ravelli Center for Neurotechnology and Experimental Brain Therapeutics, Department of Health Sciences, International Medical School, University of Milan, Milano, Italy; 4 ASST Santi Paolo e Carlo, San Paolo University Hospital, Milano, Italy; 5 IRCCS Ca’ Granda Foundation Maggiore Policlinico Hospital, Milan, Italy; 6 Department of Pathophysiology and Transplantation, “Dino Ferrari Center”, Università degli Studi di Milano, Milano, Italy

**Keywords:** Edinburgh Cognitive and Behavioural ALS Screen, Amyotrophic lateral sclerosis, Frontotemporal degeneration, Behavioural symptom, Psychometrics

## Abstract

**Background:**

This study aimed at providing diagnostic properties and normative cut-offs for the Italian ECAS Carer Interview (ECAS-CI).

**Materials:**

*N* = 292 non-demented ALS patients and *N* = 107 healthy controls (HCs) underwent the ECAS-CI and the Frontal Behavioural Inventory (FBI). Two ECAS-CI measures were addressed: (1) the number of symptoms (NoS; *range* = 0–13) and (2) that of individual symptom clusters (SC; *range* = 0–6). Diagnostics were explored against an FBI score ≥ than the 95th percentile of the patients’ distribution.

**Results:**

Both the NoS and SC discriminated patient from HCs. High accuracy, sensitivity, and specificity were detected for both the NoS and SC; however, at variance with SC, the NoS showed better post-test features and did not overestimate the occurrence of behavioural changes. The ECAS-CI converged with the FBI and diverged from the cognitive section of the ECAS.

**Discussion:**

The ECAS-CI is a suitable screener for behavioural changes in ALS patients, with the NoS being its best outcome measure (cut-off: ≥ 3).

## Background

The Edinburgh Cognitive and Behavioural ALS Screen (ECAS) [1] is the current gold-standard option to screen for cognitive and behavioural changes in ALS patients [2–4]. Although its cognitive section has been thoroughly explored as to its psychometrics, diagnostics and feasibility [3, 4], its behavioural one, i.e. the ECAS Carer Interview (ECAS-CI), has been to day neglected. The ECAS-CI is a short-lived, 13-item, caregiver-report questionnaire covering the major frontotemporal-*spectrum* behavioural features typical of ALS patients, i.e. disinhibited traits, apathetic features, reduced sympathy/empathy, obsessive–compulsive-*spectrum* symptoms and alterations in eating behaviour [1].

Given the prognostic relevance of behavioural changes in ALS, also with respect to caregivers’ burden [5], the availability of diagnostically sound, disease-specific behavioural instruments is clinically pivotal [3, 4]. Although a number of ALS-specific, behavioural instruments are currently available in Italy [6], a short-lived scale such as the ECAS-CI has not been addressed to this day within this Country. In addition, the ECAS-CI has never undergone a formal standardization within the international literature [3, 4].

Give the above premises, this study aimed at delivering diagnostic properties and normative cut-offs for the Italian ECAS-CI in ALS patients.

## Methods

### Participants

*N* = 292 consecutive, clinically diagnosed ALS patients [7] referred to IRCCS Istituto Auxologico Italiano, Milano, Italy, were recruited. Patients were non-demented, as not meeting either Rascovsky et al.’s [8] or Gorno-Tempini et al.’s [9] criteria for behavioural variant-frontotemporal dementia (bvFTD) and progressive non-fluent aphasia/semantic dementia, respectively. In addition, *N* = 107 healthy controls (HCs) were recruited via authors’ personal acquaintances. Exclusion criteria, applying to both patients and HCs, were (1) (ALS-unrelated) neurological/psychiatric diagnoses; (2) general-medical conditions possibly entailing encephalopathic features (i.e. system/organ failures or severe, uncompensated metabolic/internal diseases); and (3) uncorrected hearing/vision deficits.

### Materials

Participants underwent the Italian versions of the ECAS-CI [10] and Frontal Behavioural Inventory (FBI) [11]. Both the ECAS-CI and the FBI were administered in-person to patients’ caregivers or HCs’ family members. Additionally, patients also underwent the cognitive section of the Italian ECAS [10].

Two ECAS-CI measures were addressed: (1) the number of symptoms (NoS; *range* = 0–13, including also those 3 assessing psychotic features) and (2) that of individual symptom clusters (SC), i.e. disinhibition, apathy, loss of sympathy/empathy, perseveration, altered eating habits and psychosis (*range* = 0–6). The NoS measure is scored based on the total number of specific behavioural symptoms present (out of 13), whereas the SC one based on the presence of at least one behavioural feature within each of the 6 domains addressed by the ECAS-CI.

### Statistics

Diagnostics were explored via ROC analyses against an FBI score ≥ than the 95th percentile of the patients’ distribution. Sensitivity, specificity, positive and negative predictive values and likelihood ratios were computed at the optimal cut-off identified via Youden’s index. The minimum sample size was estimated, by means of *easyROC* (http://www.biosoft.hacettepe.edu.tr/easyROC/) [12], at *N* = 82 by addressing an allocation ratio of 1, AUC = 0.7, α = 0.05 and 1-β = 0.95. Cohen’s *k* was subsequently adopted to test the agreement rate between the classifications yielded by the two ECAS-CI measures.

ECAS-CI measures did not distribute normally (i.e. skewness ≥|1 and kurtosis ≥|3|) [13]; hence, construct validity against the FBI and the total score on the ECAS cognitive section was tested via Bonferroni-corrected Spearman’s correlations.

The effect of confounders (i.e. age, education, sex, *C9orf72* mutation, disease duration and bulbar, respiratory, upper- and lower-limb ALSFRS-R subscores) on ECAS-CI measures was tested via negative binomial regressions, which allow to model right-skewed and overdispersed count data [14] and have been previously proved effective in analysing quantitative, behavioural outcomes of ALS patients [15]. Within such models, the threshold for significance testing was derived via Bonferroni’s correction as follows: *α*_adjusted_ = 0.05/*k*, with *k* being the total number of independent variables entered into the models themselves.

The same statistical approach was employed in order to determine whether ECAS-CI measures were able to discriminate patients from HCs; within such a model, age, education and sex were covaried since the two groups were not matched for such demographics.

Analyses were run with jamovi 2.3 (https://www.jamovi.org/); the significance level (*α* = 0.05).

## Results

Table 1 reports patients’ background and clinical features. 5.8% of patients had an FBI score ≥ than the 95th percentile of the sample (i.e. ≥ 11).

**Table 1:** Patients’ background and clinical features

	ALS	HCs	*p*
*N*	292	107	-
Age (years)	64.1 ± 11.3 (20–88)	53.9 ± 10.9 (24–86)	< .001^a^
Sex (M/F)	59.9%/40.1%	36.4%/64.6%	< .001^b^
Education (years)	11.3 ± 4.4 (5–24)	13.2 ± 4 (5–22)	< .001^a^
Disease duration (months)	18.3 ± 17 (2–120)	-	-
ALSFRS-R	37.4 ± 6.8 (12–48)	-	-
KSS			
Stage 0	1.5%	-	-
Stage 1	32.2%	-	-
Stage 2	33.7%	-	-
Stage 3	26.1%	-	-
Stage 4	6.5%	-	-
PEG	0.4%	-	-
NIV	5.8%	-	-
Genetics			
*C9orf72*	6.8%	-	-
*SOD1*	2.7%	-	-
*TARDBP*	3.4%	-	-
*FUS*	0.3%	-	-
ECAS – Cognitive section			
Total	98.9 ± 19.2 (31–129)	-	-
ALS-specific	72.9 ± 15.7 (21–97)	-	-
ALS-nonspecific	26.1 ± 5.2 (9–34)	-	-
ECAS Carer Interview			
Number of symptoms (/13)	0.7 ± 1 (0–5)	0.1 ± 0.3 (0–3)	< .001^c^
Symptom clusters (/6)	0.7 ± 0.9 (0–4)	0.1 ± .3 (0–3)	< .001^c^
Percentage of behavioural changes			
Disinhibition	6.8%	0.02%	-
Loss of sympathy/empathy	16.4%	0.009%	-
Apathy	35.3%	-	-
Perseveration	2.1%	0.009%	-
Altered eating behaviour	3.8%	0.009%	-
Psychosis	2.1%	-	-
FBI	3.3 ± 3.8 (0–21)	0.7 ± 2.2 (0–16)	< .001^c^

*ALS*, amyotrophic laterals sclerosis; *ALSFRS-R*, Amyotrophic Lateral Sclerosis Functional Rating Scale-Revised; *ECAS*, Edinburgh Cognitive and Behavioural ALS Screen; *F*, female; *FBI*, Frontal Behavioural Inventory; *KSS*, King’s staging system; *M*, male; *NIV*, non-invasive ventilation; *PEG*, percutaneous endoscopic gastrostomy. ^a^Welch *t* statistic (independent sample *t* test); ^b^χ^2^ statistic (χ^2^ test of independence); ^c^χ^2^ statistic (negative binomial regression; comparison covaried for age, education and sex)

The two ECAS-CI measures showed optimal and comparable accuracy, sensitivity and specificity; however, post-test diagnostics were better for the NoS than SC (Table 2). Based on cut-offs herewith derived, 6.8% of patients presented with behavioural changes according to NoS (≥ 3), whereas 16.8% according to SC (≥ 2). A 90% agreement between the two ECAS-CI measures was found (Cohen’s *k* = 0.53; *z* = 10.3; *p* < 0.001), with disagreements being accounted for by 29 patients classified as presenting with behavioural changes by the NoS but not by SC.

**Table 2:** Diagnostic properties of the Italian ECAS-Carer Interview

	AUC	Cut-off (*J*)	Se	Sp	PPV	NPV	LR +	LR −
ECAS Carer Interview								
Number of symptoms	.92	≥ 3 (.68)	70.6%	97.1%	60%	98.2%	24.35	.3
Symptom clusters	.91	≥ 2 (.63)	76.5%	86.9%	26.5%	98.4%	5.84	.27

*ALS*, amyotrophic lateral sclerosis; *AUC*, area under the curve; *ECAS*, Edinburgh Cognitive and Behavioural ALS Screen; *LR* + , positive likelihood ratio; *LR − *, negative likelihood ratio; *J*, Youden’s index; *PPV*, positive predictive value; *NPV*, negative predictive value; *Se*, sensitivity; *Sp*, specificity

With regard to construct validity analyses, at α_adjusted_ = 0.025, both the NoS and SC correlated with the FBI (*r*_*s*_(292) ≥ 0.67; *p* < 0.001), whilst not with the total score on the ECAS cognitive section (*p* ≥ 0.215).

Negative binomial regressions for testing the effect of confounders revealed that, at α_adjusted_ = 0.006, no demographic or clinical feature were predictive of either the NoS (*p* ≥ 0.02) or SC (*p* ≥ 0.03) within the patient cohort.

As to case–control discrimination analyses, net of age, education and sex, HCs scored lower than patients on both the NoS and SCs (Table 1).

## Discussion

The present study provides, for the first time, diagnostic information and normative cut-offs for the ECAS-CI in ALS patients—delivering such information for Italian practitioners and clinical researchers.

Overall, both ECAS-CI measures herewith addressed, namely, the NoS and SC, showed adequate psychometric and diagnostic properties.

Nevertheless, albeit both the NoS and SC substantially agreed in classifying patients and showed similar accuracy and intrinsic diagnostics, the NoS yielded better post-test features and, at variance with SC, did not overestimate the occurrence of behavioural changes, being also more consistent with the prevalence yielded by the gold-standard herewith addressed (i.e. the FBI). Hence, the NoS, and not SC, should be adopted as the outcome measure of the ECAS-CI in both clinical practice and research.

Moreover, the ECAS-CI showed both convergent and divergent validity, discriminated patients from HCs and was independent of demographic and clinical confounders—this last feature supporting its adoption regardless of patients’ clinical presentation.

The ECAS-CI adds up and complements the currently available range of disease-specific/-nonspecific, behavioural tools that have been standardized in Italy within the ALS population, namely, the Emotional Lability Questionnaire (ELQ) [16], the Dimensional Apathy Scale [17], the State- and Trait-Anxiety Inventory-Form Y (STAI-Y) [18], the ALS Depression Inventory-12 (ADI-12) [19] and the Beaumont Behavioural Inventory (BBI) [15]. However, at variance with them, the ECAS-CI is less time-consuming, as well as embedded within a full, ALS-specific cognitive and behavioural screener, whose section that assesses cognition has been extensively examined for its clinimetrics and feasibility in Italy, i.e. the ECAS [10, 20, 21]. Therefore, the ECAS-CI appears to be suitable for providing, within time-restricted settings, preliminary information on patients’ behavioural status, which could be later assessed more in-depth, if useful, with the abovementioned, more specific/extensive scales [6]. After all, the ECAS-CI covers the key behavioural features of bvFTD listed within Rascovsky et al.’s [8] criteria, on which Strong et al.’s [2] classification system in turn relies for the diagnoses of ALS with behavioural impairment (ALSbi) and ALS with bvFTD. Nevertheless, it has to be borne in mind that an above-cut-off ECAS-CI alone (i.e. NoS ≥ 3) does not necessarily imply a diagnosis of ALSbi pursuant to Strong et al.’s [2] criteria, being rather an index of FTD-like behavioural changes in general: by contrast, such a nosographic categorization is up to the examiner and has to be based on which behavioural symptom(s), among those listed within the ECAS-CI; the examinee is positive to. For instance, according to Strong et al.’s [2] criteria, the detection of apathetic features alone would be sufficient for classify a patients as ALSbi.

The main limitations of this work lie in (1) the adoption of an ALS-nonspecific gold-standard (i.e. the FBI); (2) the low prevalence of FBI-detected behavioural changes, with this last aspect having possibly biased prevalence-based diagnostics (i.e. PPV and NPV); and (3) the lack of assessment of test–retest and inter-rater reliability for the ECAS-CI, which are both psychometric properties highly relevant to clinical practice and research. Additionally, two further critical elements then need to be addressed: (1) HC inclusion was herewith solely based on medical history, without specific psychometric assessment of cognition or psychopathology and (2) patients were not stratified according to Strong et al.’s [2] criteria, as this last element being beyond the scope of the present investigation. However, the agreement rate between nosographic systems [2] and the ECAS-CI, at least with regard to those diagnoses involving behavioural changes, should be addressed within future investigations.

In conclusion, the ECAS-CI is a suitable screener for behavioural changes in ALS patients, which can be informative towards the need for administering more specific/extensive, behavioural/psychological scales currently available in Italy [6]. Data herewith presented will also allow clinicians and researchers exploiting the ECAS beyond its cognitive section [10, 20–22], considering the crucial entailments of behavioural changes towards patients’ prognosis, clinical management and family caregiving [23].

## Data Availability

Datasets associated with the present study are accessible at the following link (upon request to the Corresponding Author): https://zenodo.org/record/7347119#.Y3zRDXbMJPZ.
